# Sex differences in noradrenergic regulation of the medial prefrontal cortex in mice

**DOI:** 10.1186/s13293-025-00779-4

**Published:** 2025-11-12

**Authors:** Marcis V. Scroger, Alexandria C. Athanason, Noah M. Paperny, Andrea Liss, Katie T. Vo, Misha Muneeb, Mahum T. Siddiqi, Molly R. Batchelder, Iman Shahbaz, Serena Chan, Molly M. Deak, Anushree N. Karkhanis, Florence P. Varodayan

**Affiliations:** 1 Developmental Exposure Alcohol Research Center and Behavioral Neuroscience Program, Department of Psychology, Binghamton University-SUNY, Binghamton, NY USA; 2 Department of Psychology, Binghamton University-SUNY, 4400 Vestal Parkway East, S4-175G,, Binghamton, NY 13902, USA

**Keywords:** Anxiety, Cognitive, Excitation, Female, Glutamate, Locus coeruleus, Noradrenaline, Norepinephrine, Spontaneous excitatory postsynaptic currents (sEPSC), Stress, Synaptic transmission

## Abstract

**Background:**

Norepinephrine (noradrenaline; NE) is a stress signal released from the locus coeruleus (LC) into the prefrontal cortex (PFC) to govern arousal, attention, and cognition. The LC is sexually dimorphic, and PFC NE dysfunction contributes to alcohol use disorder and several stress-related neuropsychiatric disorders that manifest differently in men and women. However, most preclinical studies of the medial PFC (mPFC) NE system have only used male subjects. Additionally, even though each mPFC subregion and layer forms unique circuits that mediate different aspects of cognitive behavior, their specific neuromodulation by NE is not understood.

**Methods:**

We comprehensively probed potential sex differences in the mouse mPFC NE system, starting with fluorescent tracing of the LC→mPFC circuit. Basal mPFC NE tissue content and adrenergic receptor mRNA were measured using high performance liquid chromatography and real-time quantitative polymerase chain reaction. *Ex vivo* electrophysiology assessed NE modulation of glutamate synapses in layers 2/3 and 5 of the prelimbic and infralimbic subregions of the mPFC. Finally, we used an α2 adrenergic receptor antagonist to increase NE release and tested for mPFC-associated reversal learning and episodic memory.

**Results:**

Females had a greater percentage of LC NE neurons→mPFC than males, with no differences in basal mPFC NE concentration or adrenergic receptor mRNA. NE increased mPFC glutamate release broadly in males, but its effects in females were restricted to prelimbic layer 5 and infralimbic layer 2/3. Finally, while there were dose-dependent effects of the α2 receptor antagonist on cognitive behavior, they did not vary between sexes.

**Conclusions:**

We uncovered complex sex differences in LC→mPFC structure and mPFC NE function, and future studies should examine NE activation in the context of greater cognitive load, such as during alcohol withdrawal or periods of stress. Clinically, women exhibit greater stress-induced activation of the NE system, are more likely to be diagnosed with affective disorders, and are more likely to drink alcohol to regulate negative affect and stress reactivity than men. Therefore, our study highlights the importance of considering specific subpopulations (e.g. women, or people with comorbid stress and alcohol use disorders) during the development of new NE-based treatments.

**Plain english summary:**

Norepinephrine (also known as noradrenaline) is a stress signal that regulates activity in the brain region the medial prefrontal cortex (mPFC) to optimize decision making, emotional processing, inhibitory control, and learning and memory. Its dysfunction contributes to both alcohol use disorder and stress-related neuropsychiatric disorders, though its role may differ between men and women. It is well known that the brain region that makes norepinephrine (i.e. locus coeruleus; LC) is larger and more complex in women and female rodents than their male counterparts. However, most preclinical studies have only used male subjects so the impact of these sex differences remains unclear. In this study, we used male and female mice to probe the LC→mPFC brain circuit and understand how norepinephrine shapes mPFC neuronal communication. We also tested how increasing norepinephrine levels alters cognitive behaviors that are mediated by the mPFC. We identified complex sex differences; female mice had a larger LC→mPFC circuit but their mPFC neurons were less sensitive to norepinephrine compared to male mice. There were no sex differences in norepinephrine’s effects on reversal learning and episodic memory under baseline conditions, but future studies should examine whether sex differences emerge during alcohol withdrawal or periods of stress. This work expands our understanding of mPFC norepinephrine signaling in both sexes and highlights the importance of considering specific subpopulations (e.g. women, or people with comorbid stress and alcohol use disorders) during the development of new norepinephrine-based treatments.

**Highlights:**

- Females have a larger locus coeruleus → medial prefrontal cortex circuit than males.
- Basal norepinephrine levels and adrenergic receptor gene expression levels are similar in the medial prefrontal cortex of male and female mice.
- Norepinephrine broadly increases glutamate release onto medial prefrontal cortex pyramidal neurons in male mice.
- In female mice, norepinephrine-induced glutamate release is restricted to prelimbic cortex layer 5 and infralimbic cortex layer 2/3 pyramidal neurons.
- Norepinephrine altered medial prefrontal cortex-dependent cognitive behaviors (reversal learning and episodic memory) in mice, but there were no sex differences in its effects.

## Background

Norepinephrine (noradrenaline; NE) is a major brain stress signal that regulates arousal, attention and cognition through top-down control of subcortical brain regions [1, 2]. It is predominantly released from the locus coeruleus (LC) across the forebrain, including into the dorsolateral prefrontal cortex (dlPFC) of humans and non-human primates and into the medial prefrontal cortex (mPFC) of rodents. The dl/mPFC can be divided into subregions that send excitatory glutamatergic projections to overlapping and distinct subcortical brain regions; in rodents, pyramidal neurons in superficial layer 2/3 of both the prelimbic and infralimbic mPFC send outputs to the basolateral amygdala, ventral striatum, and contralateral mPFC, while deeper layer 5 pyramidal neurons project to the lateral hypothalamus, lateral habenula and midline/medial thalamic nuclei (including the paratenial nucleus, nucleus reuniens and mediodorsal nucleus) [3–6]. Prelimbic layer 5 neurons also preferentially target the nucleus accumbens and insula cortex, while infralimbic layer 5 neurons send outputs to the bed nucleus of the stria terminalis and lateral septum [6]. The effects of NE on mPFC function are not well delineated across subregions/layers, though there is evidence that prelimbic NE mediates fear-conditioning and drug-seeking expression and consolidation, while infralimbic NE regulates their extinction [7–16].

Successful execution of these mPFC-dependent behaviors relies on both the local NE concentration as well as the activity of each class of adrenergic receptors (AR; α1R, α2R, and βR). LC neurons have two action potential firing modes, tonic (0.1–5 Hz) and phasic (short bursts of 10–20 Hz), that drive mPFC NE release, and AR differ based on their affinity for NE (α2R >α1R >βR) and intracellular signaling (α2R/G_i_-, α1R/G_q_-, or βR/G_s_-coupled) [2, 17]. Adding to this complexity, the density of NE varicosities and AR subtype expression have been found to vary across mPFC layers [2, 18]. Importantly, α2 autoreceptors are also expressed on noradrenergic presynaptic terminals where they create a negative feedback loop to shut down excessive NE release. While mPFC pyramidal cell excitability is generally increased by NE/α1R or NE/βR signaling and decreased by NE/α2R activity [10, 19–28], though see [29–32], the subregion- and layer-specific effects of NE on pyramidal neurons have not been systematically delineated. Overall, it is proposed that tonic LC firing provides the mPFC with a relatively low amount of NE/α2R signaling to mediate basal function during relaxed states [2]. In periods of focused attention or stress (i.e. perceived threat), the LC switches to phasic firing. This increases NE release so it can bind to a subset of α1R, ultimately leading to filtering of irrelevant task stimuli and optimal cognitive function. Prolonged stress can further increase mPFC NE, triggering high levels of β1R and α1R activation, leading to deficits in working memory, attention, and impulse control. This inverted U-shaped curve of noradrenergic influence over cognitive function (reflecting the Yerkes-Dodson law) has important implications for our understanding of neuropsychiatric diseases that involve mPFC noradrenergic dysfunction, including alcohol use disorder, chronic stress, and anxiety disorders [2, 33].

Critically, most of the studies establishing mPFC NE/AR patterning and its behavioral outcomes were conducted using only male subjects. This is particularly surprising as women and female rodents have larger LC than their male counterparts, with more NE neurons and more complex dendrites resulting in more peri-LC synaptic contacts [1]. Whether this sexual dimorphism translates to differences in mPFC NE activation, potentially shifting vulnerability to stress-induced cognitive impairment, remains unknown. Therefore, here we used C57BL/6J mice to compare the male and female LC→mPFC circuit, characterize mPFC NE signaling in a sex-, subregion- and layer-specific manner, and probe NE’s sex-specific influence over cognitive function.

## Methods

### Animals

Adult male and female C57BL/6J mice (*N* = 273; shipped at 10–12 weeks old) were obtained from The Jackson Laboratory (Bar Harbor, ME), and underwent a minimum 1-week acclimation period. All mice were group-housed in a temperature- and humidity-controlled room on a 12-h reverse light cycle (dark period was 10:00 am–10:00 pm) for the duration of the experiment, and food and water were available *ad libitum*. Female mice cycled freely in all experiments. Of note, separate cohorts of mice were used for studying each of the following: (1) LC→mPFC circuit tracing, (2) mPFC basal NE tissue concentration, (3) mPFC AR gene expression, (4) NE regulation of mPFC glutamate transmission, and (5) NE regulation of each cognitive and anxiety-like behavior. All experimental procedures were approved by Binghamton University – SUNY Institutional Animal Care and Use Committee, consistent with the National Institutes of Health Guide for the Care and Use of Laboratory Animals.

### Fluorescent circuit tracing

*Surgery*.

Mice (*N* = 12–13 mice/sex) were pre-emptively administered analgesics (subcutaneous 0.05–0.1 mg/kg of buprenorphine hydrochloride, 2.5–5.0 mg/kg of carprofen, and 1–2 mg/kg of 0.25% bupivacaine without epinephrine), deeply anesthetized with isoflurane (3–5% induction, 1–3% maintenance) and placed into the stereotaxic frame. The skull was exposed, 1 mm holes were drilled into the skull, and a 26-gauge stainless steel injector was inserted. Retrograde 10 kDa fluorescein-tagged dextran (0.5 uL) was bilaterally injected into the medial prefrontal cortex (2.1 AP, +/−0.4 ML, 1.4 DV from Bregma) [34, 35] at a rate of 0.15 µL/min via a 10 µL Hamilton syringe mounted on a Microdrive pump (Harvard Apparatus). Following tracer delivery, the needle was left in place for 10 min. The incision site was closed with sutures. Immediately following surgery, mice were placed on a heating pad in a separate recovery cage and monitored for a minimum of 1 h. Mice underwent a three-week recovery period to permit tracer transport.

*Immunofluorescence & microscopy*.

Mice were administered Fatal-Plus (150 mg/kg *i.p*.), and then underwent transcardial perfusion using 15 mL of 0.1 M phosphate buffered saline (PBS) followed by 15 mL of 4% paraformaldehyde (PFA) in PBS (pH 7.4) between 10:00–5:00 pm [36]. Brains were then extracted and post-fixed in 4% PFA at 4 °C overnight, followed by 30% sucrose in PBS cryoprotectant solution at 4 °C for 3 days. Brains were then flash frozen in 2-methylbutane on dry ice for 2 min and stored at −20 °C. Free-floating LC and mPFC slices were coronally sectioned (40 μm) using the cryostat and stored in antifreeze at −20 °C.

To label putative LC NE neurons, bilateral sections containing the LC were triple-rinsed in 0.1 M PBS (5 min each rinse) and incubated in blocking buffer (5% normal donkey serum, 1% bovine serum albumin, 22.52 mg/mL glycine, 0.2% Triton X-100, 0.1 M PBS) at room temperature for 1 h. Sections were then incubated in rabbit dopamine β-hydroxylase (DβH) polyclonal antibody (1:400, ThermoFisher, PA5-34664) diluted in a blocking buffer at 4 °C overnight. After 3 PBS rinses (5 min each), sections were incubated with a donkey anti-rabbit AF594 fluorescent dye-conjugated secondary antibody (1:1000, ThermoFisher, A-21207) diluted in blocking buffer at room temperature for 1 h. After 3 PBS rinses (5 min each), sections were mounted on gelatin-coated slides and cover-slipped with 150 uL of Prolong Gold Antifade Mountant with DAPI (Invitrogen).

Microscopy images (Olympus Research Slide Scanner VS200, 20x magnification) were taken to visualize the LC. Of note, mPFC injection site and spread could not be verified at this 3-week timepoint due to efficient fluorescein-tagged dextran axonal uptake and transport. The borders of the LC were defined by the clusters of DβH-immunoreactive neurons located ventrolateral to the fourth ventricle and directly adjacent to the mesencephalic trigeminal tract in the AP range -5.34 to -5.52 mm relative to Bregma, consistent with the coordinates reported in [35]. ImageJ software [37] was used to manually count the total number of putative NE neurons (DβH+) and the number of putative NE neurons that project to the mPFC (DβH+ and fluorescein-tagged dextran+) in each LC section. Four LC sections were averaged per mouse, with the data independently collected by two lab members blinded to the sex of the mouse.

### NE tissue content

Total mPFC NE tissue content was quantified under basal conditions using high pressure liquid chromatography (HPLC). Briefly, mice (*N* = 19–20 mice/sex) were anesthetized with 3–5% isoflurane and decapitated between 11:00–1:00 pm [38]. Brains were extracted and cut into 1 mm thick sections with a cooled brain matrix. Bilateral micropunches (1 mm) of the mPFC were collected in pre-weighed tubes, immediately placed on dry ice and then stored at −80 °C. Tissue was suspended in 0.2 M HClO_4_ (20 µL per 1 mg of tissue), sonicated, and centrifuged for 15 min at 12,000 rpm at 4 °C. Supernatant was analyzed using HPLC with electrochemical detection (SenCell with 2 mm GC WE, AST position 1, DECADE Lite, ANTEC, Netherlands). Specifically, 8 µL of each sample was injected into a reverse-phase microbore column (Acquity UPLC BEH C18, 1.7 μm, 1 × 100 mm, Waters, Milford, MA) for separation. The following mobile phase was used for NE detection: 100 mM phosphoric acid, 100 mM citric acid, 0.1 mM EDTA-Na_2_ set to pH 3.0, 600 mg/L octanesulfonic acid sodium salt, and 8% acetonitrile. NE standards (20, 10, 5, 2, 1 nM) were used, and data are reported as NE concentration per 1 µL of sample.

### Gene expression

mPFC AR gene expression was assessed in a separate cohort of mice (*N* = 10 mice/sex) using real time reverse transcription polymerase chain reaction (RT-PCR). Mice were anesthetized with 3–5% isoflurane and decapitated between 11:00–1:00 pm, and brains extracted and immediately flash frozen in 2-methylbutane then stored at −80 °C [39–41]. A 5 mm stainless steel bead and a TissueLyser (both Qiagen, Valencia, CA) were used to homogenize each pair of mPFC bilateral micropunches (1.20 mm) in Trizol reagent (Sigma-Aldrich, St. Louis, MO). Qiagen RNeasy columns were used to extract total RNA, which was then analyzed for concentration and purity using a Nanodrop spectrophotometer (Themoscientific, Waltham, MA). The Qiagen QuantiTect Reverse Transcription kit (Cat. No. 205313) was used for cDNA synthesis. The CFX384 real-time PCR detection system, iQ SYBR Green Supermix (Biorad, Hercules, CA), cDNA template and primers specific to each target gene (indicated by a single peak in the melt curve; Table 1) were used for RT-PCR. The reference gene glyceraldehyde-3-phosphate dehydrogenase (*Gapdh: t*(16) = 0.40, *p* = 0.69) was used for normalization using the ΔΔCt method, with the male group arbitrarily designated as the ultimate control. The ROUT test (Q = 1%) was used to remove any outliers from a specific gene’s analysis.

**Table 1:** Primers pairs used for gene expression study

Gene name	Gene symbol	Accession number	Forward primer	Reverse primer
Glyceraldehyde 3-phosphate dehydrogenase	*Gapdh*	NM_008084.3	AGGTCGGTGTGAACGGATTT	TGCCGTGAGTGGAGTCATAC
Adrenergic receptor, α1_A_ variants 1–3	*Adra1a v1-3*	NM_001271761.1	CCGTGAGGCTGCTCAAGTTT	AAATTCGGGAAGAAGGACCCAAT
Adrenergic receptor, α1_A_ variants 1,2,4	*Adra1a v1*,*2*,*4*	NM_001271761.1	GACTGGGTCTTGGTCTTTGGA	GGCCCTGGAGCTTCGTTT
Adrenergic receptor, α1_B_	*Adra1b*	NM_001284381.1	ACCTTGGGCATTGTAGTCGG	GGAGAACAGGGAGCCAAGTG
Adrenergic receptor, α1_D_	*Adra1d*	NM_013460.5	TCTCCGTAAGGCTGCTCAAG	GAGGGAACAGAGAACCCAGAG
Adrenergic receptor, β1	*Adrb1*	NM_007419.3	CTGCTACAACGACCCCAAGT	CACGTAGAAGGAGACGACGG
Adrenergic receptor, β2	*Adrb2*	NM_007420.3	AATAGCAACGGCAGAACGGA	TCAACGCTAAGGCTAGGCAC

### Electrophysiology

Mice (*N* = 24–27 mice/sex) were anesthetized (3–5% isoflurane) and decapitated between 8:00–2:00 pm, and their brains were extracted into oxygenated (95% O_2_/5% CO_2_), cold high-sucrose solution (pH 7.3–7.4): 206.0 mM sucrose; 2.5 mM KCl; 0.5 mM CaCl_2_; 7.0 mM MgCl_2_; 1.2 mM NaH_2_PO_4_; 26.0 mM NaHCO_3_; 5.0 mM glucose; 5.0 mM HEPES [36, 41, 42]. Brain tissue containing the mPFC was coronally sliced (300–400 μm; Leica VT1200S vibratome, Buffalo Grove, IL) and incubated in oxygenated artificial cerebrospinal fluid (aCSF): 130 mM NaCl, 3.5 mM KCl, 2 mM CaCl_2_, 1.25 mM NaH_2_PO_4_, 1.5 mM MgSO_4_, 24 mM NaHCO_3_, and 10 mM glucose for at least 1 h at 32 °C.

For each whole-cell voltage-clamp recording, slices were perfused with 2 mL/min room-temperature oxygenated aCSF, and neurons were visualized with infrared-differential interference contrast (IR-DIC) optics, a 40x water immersion objective (Olympus BX51WI, Tokyo, Japan) and a Retiga electro CCD camera (Teledyne, Thousand Oaks, CA). mPFC layer 2/3 and layer 5 pyramidal neurons were located 100–300 μm and 350–500 μm from the pial surface, respectively, and identified by their characteristic size and shape. Recordings were collected with gap-free acquisition mode and a low-pass filter (10 kHz), using a Multiclamp 700B amplifier, Digidata 1550A and pClamp 10.2 software (all Molecular Devices, Sunnyvale, CA) from cells clamped at −70 mV.

Potassium gluconate internal solution: 135 mM K^+^-gluconate, 5 mM EGTA, 5 mM MgCl_2,_ 10 mM HEPES, 2 mM Mg^2+^-ATP, 0.2 mM Na^+^-GTP was used to fill 4 –7 MΩ pipettes, and spontaneous glutamatergic excitatory postsynaptic currents (sEPSCs) were pharmacologically isolated using 1 µM CGP 55845A and 30 µM bicuculline. A 10-mV pulse was used to monitor series resistance, and recordings with a series resistance > 25 MΏ or a > 20% change in series resistance were excluded. All drugs were applied directly to the bath in known concentrations. CGP 55845A and NE were purchased from Tocris Biosciences (Ellisville, MI) and bicuculline from Sigma (St. Louis, MO). No more than one cell per mouse was used for each experimental group.

The sEPSC frequency, amplitude, rise time and decay time were analyzed using Mini Analysis (Synaptosoft Inc., Fort Lee, NJ) and visually confirmed over an 85–180 s interval at baseline and within 6–15 min of NE application. Of note, the experimenter was not blinded to the mouse group information, but the recordings were analyzed in a blinded manner. To control for variation in baseline electrophysiology properties across cells, NE effects were normalized to their own neuron’s baseline prior to group analyses. Generally, increased sEPSC frequencies are indicative of higher release probabilities, while changes in amplitude and kinetics are associated with altered postsynaptic receptor function [43]. The ROUT test (Q = 1%) was used to identify any sEPSC outliers, and that entire cell was removed from the experiment. Specifically, 1 cell was removed from the male PL2/3 sEPSC group, 1 cell from male PL5, 1 cell from male IL5 and 2 cells from female IL5.

There were no sex differences in baseline PL and IL layer 2/3 and 5 sEPSCs (Table 2).

**Table 2:** Baseline sEPSC properties in mPFC pyramidal neurons. sEPSC frequencies, amplitudes, rise times and decay times (prior to any NE application) of pyramidal neurons from layers 2/3 and 5 of the prelimbic and infralimbic mPFC of male and female mice. There were no sex differences in baseline sEPSCs in PL layer 2/3 [*freq: t*(18) = 1.50, *p* = 0.15; *amp: t*(18) = 1.46, *p* = 0.16; *rise: t*(18) = 0.26, *p* = 0.80; *decay: t*(18) = 1.19, *p* = 0.25 by unpaired *t*-test], PL layer 5 [*freq: t*(18) = 0.011, *p* = 0.99; *amp: t*(18) = 0.83, *p* = 0.42; *rise: t*(18) = 1.81, *p* = 0.088; *decay: t*(18) = 0.31, *p* = 0.76], IL layer 2/3 [*freq: t*(18) = 1.30, *p* = 0.21; *amp: t*(18) = 1.47, *p* = 0.16; *rise: t*(18) = 0.17, *p* = 0.86; *decay: t*(18) = 0.78, *p* = 0.45], and IL layer 5 [*freq: t*(19) = 0.33, *p* = 0.75; *amp: t*(19) = 0.11, *p* = 0.91; *rise: t*(19) = 0.44, *p* = 0.66; *decay: t*(19) = 1.33, *p* = 0.20]. All data are presented as mean ± SEM

	Treatment	Frequency (Hz)	Amplitude (pA)	Rise Time (ms)	Decay Time (ms)
**Prelimbic** **Layer 2/3**	**Male** (*n* = 10 cells from 10 mice)	1.12 ± 0.22	30.43 ± 1.22	1.31 ± 0.16	1.59 ± 0.22
	**Female** (*n* = 10 cells from 10 mice)	0.74 ± 0.14	28.43 ± 0.62	1.26 ± 0.11	1.26 ± 0.17
**Prelimbic** **Layer 5**	**Male** (*n* = 10 cells from 10 mice)	1.37 ± 0.34	29.59 ± 1.12	1.59 ± 0.085	1.36 ± 0.20
	**Female** (*n* = 10 cells from 10 mice)	1.36 ± 0.31	30.71 ± 0.77	1.28 ± 0.15	1.28 ± 0.19
**Infralimbic** **Layer 2/3**	**Male** (*n* = 10 cells from 10 mice)	0.95 ± 0.19	20.84 ± 1.09	2.38 ± 0.057	2.80 ± 0.33
	**Female** (*n* = 10 cells from 10 mice)	1.28 ± 0.16	18.23 ± 1.40	2.39 ± 0.029	2.54 ± 0.11
**Infralimbic** **Layer 5**	**Male** (*n* = 10 cells from 10 mice)	1.62 ± 0.36	21.81 ± 2.15	2.38 ± 0.045	2.94 ± 0.14
	**Female** (*n* = 11 cells from 11 mice)	1.49 ± 0.22	21.51 ± 1.70	2.35 ± 0.039	2.64 ± 0.17

### Behavior

*Drugs*.

Atipamezole blocks α2 adrenergic autoreceptor-mediated inhibition of NE release, and its systemic administration by *i.p.* injection has been shown to increase mPFC extracellular NE levels [44, 45]. Atipamezole was purchased from Tocris Bioscience (Minneapolis, MN), dissolved in physiological saline to the final injection concentration, and aliquoted and stored at −20 °C for a maximum of 1 month. All mice received at least 3 days of habituation to physiological saline intraperitoneal (*i.p.*) injections before atipamezole or saline vehicle administration, and drug conditions were counterbalanced within each behavioral cohort.

*Barnes maze (BM)*.

To investigate how NE release impacts mPFC-dependent behavioral flexibility, mice (*N* = 18 mice/sex) underwent a Barnes maze reversal task [36, 39]. Briefly, mice were randomly pre-assigned to the Control or Atipamezole groups. All mice underwent 12 daily acquisition sessions, followed by 4 daily reversal tests. 20 min before each daily reversal test, atipamezole (0.5 mg/kg *i.p.)* [46, 47] or vehicle was administered.

In this task, mice had to escape into a small dark recessed box located under 1 of 20 small holes evenly distributed along the perimeter of an elevated, brightly lit, circular open platform (San Diego Instruments, San Diego, CA). Each mouse was assigned a specific escape box location that remained unchanged across acquisition sessions, with the locations randomized across all the mice. Distal visual cues aided mouse navigation. Prior to their first acquisition session, each mouse was placed in the center of the maze, guided toward the target hole, and helped to climb into the escape box where it stayed for 1 min. The mouse then entered its first acquisition trial. For each of the 12 daily acquisition sessions, the mouse was placed within an opaque cylinder on the center of the maze for 10–20 s. Then the cylinder was lifted to start the trial. The session was ended when the mouse entered the target hole. If the mouse did not enter the target hole within 3 min, it was gently guided to it and helped to climb into the escape box. After spending 1 min in the escape box, each mouse was returned to its home cage, and the maze and escape box were cleaned with 80% ethanol. 24 h after final acquisition session, the mice began 4 days of atipamezole or vehicle saline administration (details above) followed by reversal testing under the same conditions as acquisition, except that a new target hole was assigned to each mouse (rotated 90^o^ from the acquisition location). All sessions were recorded with a ceiling-mounted video camera and analyzed with Anymaze software (Stoelting Company, Wood Dale, IL). Latency to enter the target hole and number of errors (incorrect holes visited prior to entering the target hole) were quantified. Outliers were identified using the ROUT test (Q = 1%) and removed from the entire experiment, leading to *N* = 8 Male Control, *N* = 8 Female Control, *N* = 8 Male Atipamezole and *N* = 9 Female Atipamezole mice.

*Elevated plus maze (EPM)*.

To determine whether atipamezole altered anxiety-like behavior, a separate cohort of mice (*N* = 24 mice/sex) were tested using the elevated plus maze [48]. Briefly, mice were randomly pre-assigned to the Control group or Atipamezole groups. All mice underwent 3 days of habituation to the testing room. The next day, atipamezole [0.5 mg/kg *i.p.*] or vehicle was administered and then 20 min later the mice underwent EPM.

For the EPM test, each mouse was placed in the center of the brightly-lit plus-shaped maze with 2 open arms and 2 closed arms with high walls, and allowed to explore for 5 min. The maze was cleaned with 80% ethanol between each mouse. All sessions were recorded with a ceiling-mounted video camera and analyzed with Anymaze software (Stoelting Company, Wood Dale, IL). The number of entries into each arm (when all 4 paws entered an arm), as well as the amount of time spent in each arm was quantified. The % open arm entries [number of open arm entries divided by the sum of open + closed arm entries] and % open arm time [time spent in open arm divided by the sum of time spent in open + closed arms] both reflect anxiety-like behavior. The number of closed arm entries, as well as distance traveled and mean speed, reflect locomotor activity. Finally, exploratory head dips beneath the open arm and peeps over the side of the open arm were scored by two individuals who were blinded to the mouse group information. Outliers were identified using the ROUT test (Q = 1%) and removed from the entire experiment, leading to *N* = 12 Male Control, *N* = 11 Female Control, *N* = 12 Male Atipamezole and *N* = 11 Female Atipamezole mice .

*Novel object location (NOL)*.

Finally, we probed the effect of lower doses of atipamezole on mPFC-dependent episodic memory using the novel object location task in a separate cohort of mice (*N* = 27 mice/sex) [49]. Briefly, mice were randomly pre-assigned to the Control group or one of two Atipamezole groups. All mice followed an every-other-day schedule, where mice underwent 3 days of habituation to the testing arena, 2 learning sessions, and a retention test. Mice received a physiological saline *i.p.* injection 5–10 min after learning session #1. Similarly, 5–10 min after learning session #2, mice received one of two lower doses of atipamezole [0.05 or 0.15 mg/kg *i.p.*] or vehicle.

For each of the 10-min habituation sessions, the mouse was placed in the designated release corner and allowed to explore the testing arena (40 cm x 40 cm box with a white floor and black walls; San Diego Instruments, San Diego, CA). For each 10-min learning session, 2 identical weighted objects were added to the arena with each object positioned 3 cm from the nearby walls. Each mouse was placed in the designated release corner and allowed to explore the objects for encoding. After learning session #2, the mice were administered atipamezole or vehicle saline (details above) and then underwent the 10-min retention test 3–4 h later. For the retention test, one of the objects was moved to the indicated novel location and each mouse was allowed to explore both objects. The arena and objects were cleaned with 80% ethanol between each mouse.

All NOL sessions were recorded, and the data analyzed using ANY-maze software (Stoelting Company, Wood Dale, IL). Time spent with an object was defined as the mouse’s head positioned towards the object or the mouse interacting with the object (excluding climbing on it) within the 3 cm radius of the object, and the total time each mouse spent with each object was measured for each session. To determine if the mice had an initial object location preference in the 2 learning sessions, [the difference in time spent exploring each object] was divided by [the sum of time spent exploring both objects]. For the retention test, a Discrimination Ratio (DR) was defined as [the difference in time spent exploring the Object in Novel Location and the Object in Familiar Location] divided by [the sum of time spent exploring both objects]. The DR was calculated for the first 3 min of the retention test, as well as the entire 10-min period. A positive DR indicates that the mouse spent more time with the object in the novel location, suggesting that the memory of the familiar location is intact. Total distance traveled was also measured. No outliers were removed.

### Statistical analyses

Statistical analyses and figure preparation were performed using Prism version 9.5.0 (GraphPad, San Diego, CA). Outlier testing was performed using the ROUT test (Q = 1%), and if an outlier was identified, it was removed from the entire data set. Data were then analyzed using one-sample *t*-tests with a hypothetical mean of 100, unpaired *t*-tests, and two-way ANOVAs with *post hoc* Tukey’s multiple comparisons tests where appropriate, with differences significant at *p* < 0.05. Data are represented as mean ± SEM.

## Results

### Females have a larger LC→mPFC circuit than males

**Fig. 1:**
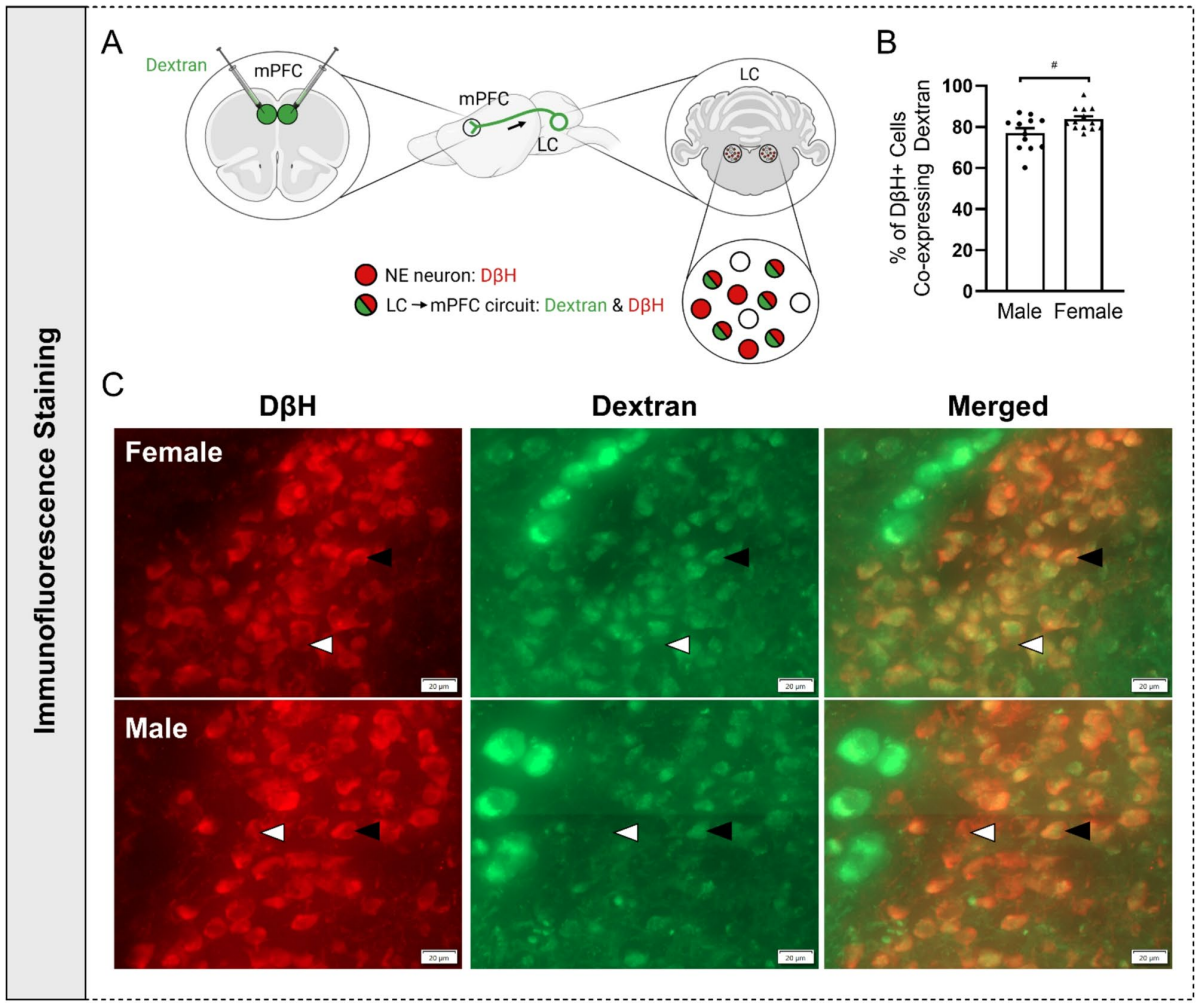
LC NE→mPFC circuit. **(A)** Schematic of intra-mPFC microinfusion of fluorescent dextran and its retrograde transport to the LC. **(B)** A greater percentage of LC NE neurons projected to the mPFC in female vs. male mice. **(C)** Representative female and male LC images from AP: −5.52 and − 5.40 mm relative to Bregma, respectively. White arrows indicate examples of putative NE neurons labeled with DβH, and black arrows indicate examples of mPFC projecting NE neurons co-labeled with dextran and DβH. *N* = 12–13 per group. ^#^*p* < 0.05 by unpaired *t*-test

A retrograde fluorescent dextran tracing approach was used to probe potential sex differences in the LC→mPFC circuit (Fig. 1A-C). Female mice had a greater percentage of LC DβH+ cells that were co-labeled with dextran compared to males, indicating a larger LC→mPFC circuit [*t*(23) = 2.462, *p* < 0.05 by unpaired *t*-test; (Fig. 1B)].

### mPFC noradrenergic patterning was similar between the sexes

**Fig. 2:**
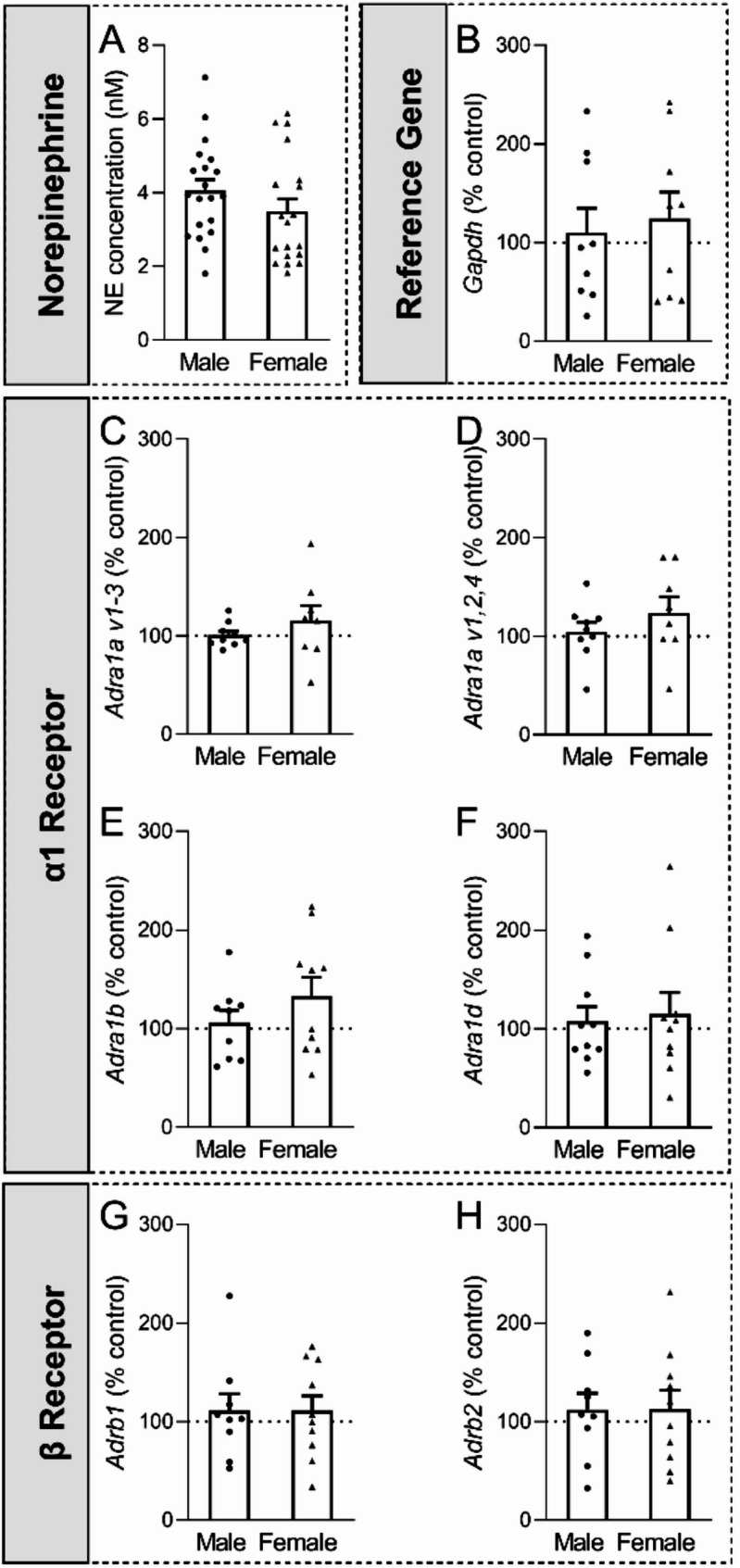
mPFC noradrenergic patterning. **A.** Male and female mPFC have similar basal levels of NE. *N* = 19–20 mice per group. **B-H.** mPFC (B) *Gapdh*, (C) *Adra1a v1-3*, (D) *Adra1a v1*,*2*,*4*, (E) *Adra1b*, (F) *Adra1d*, (G) *Adrb1*, and (H) *Adrb2* mRNA levels were comparable across both sexes. *N* = 8–10 mice per group

We next used HPLC to determine whether the size of the LC→mPFC projection impacted basal mPFC NE tissue content and we found no sex difference [*t*(37) = 1.29, *p* = 0.21; (Fig. 2A)].

In a separate cohort of mice, potential sex differences in mPFC AR gene expression were assessed using RT-PCR. mPFC α1 and β receptor mRNA levels were comparable across both sexes [*Adra1a v1-3: t*(15) = 1.03, *p* = 0.32; *Adra1a v1*,*2*,*4: t*(15) = 1.06, *p* = 0.23; *Adra1b: t*(17) = 1.15, *p* = 0.27; *Adra1d: t*(18) = 0.28, *p* = 0.79; *Adrb1: t*(17) = 0.0035, *p* = 1.00; *Adrb2: t*(17) = 0.026, *p* = 1.00; (Fig. 2B-H)].

### NE increased glutamate release across the male mPFC, but its effects were subregion- and layer-specific in females

**Fig. 3:**
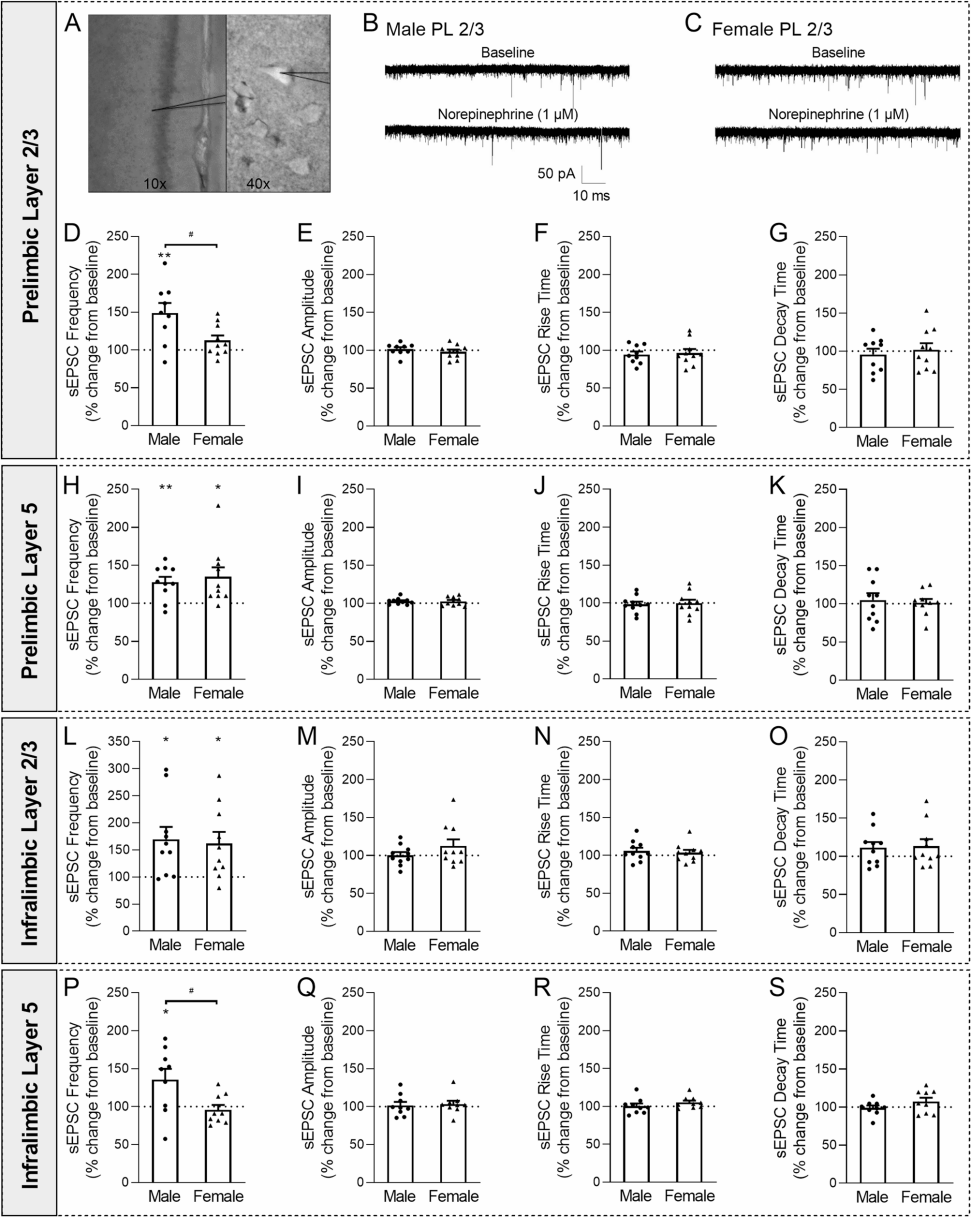
NE regulation of mPFC glutamate synapses. **A**. Micrograph of mPFC pyramidal neuron. **B-C.** Representative sEPSC traces before and during 1 µM NE application from PL layer 2/3 pyramidal neurons in (B) male and (C) female mice. **D.** NE increased the PL2/3 sEPSC frequency in male, but not female mice. **E-G.** NE had no effect on PL2/3 sEPSC (E) amplitudes, (F) rise times, or (G) decay times. **H.** NE increased the PL5 sEPSC frequencies in male and female mice. **I-K.** NE had no effect on PL5 sEPSC (I) amplitudes, (J) rise times, or (K) decay times. **L.** NE increased the IL2/3 sEPSC frequencies in male and female mice. **M-O.** NE had no effect on IL2/3 sEPSC (M) amplitudes, (N) rise times, or (O) decay times. **P.** NE increased the IL5 sEPSC frequency in male, but not female mice. **Q-S.** NE had no effect on IL5 sEPSC (Q) amplitudes, (R) rise times, or (S) decay times. *n* = 9–10 cells per group. All data are presented as mean ± SEM. ^*^p < 0.05, ^**^p < 0.01 by one-sample *t*-test with a hypothetical mean of 100. ^#^*p* < 0.05 by unpaired *t*-test

We then studied the functional impact of NE signaling on mPFC glutamate synapses using* ex vivo* electrophysiology (baseline sEPSC properties reported in Table 2). We found that 1 µM NE increased the sEPSC frequency of prelimbic mPFC layer 2/3 (PL2/3) pyramidal neurons in male mice, but had no effect in female mice [male: *t*(8) = 3.72, *p* < 0.01; female: *t*(9) = 1.90, *p* = 0.09 by one-sample *t*-test; male vs. female: *t*(17) = 2.55, *p* < 0.05 by unpaired *t*-test; (Fig. 5A-D)]. There were no effects of NE on the sEPSC amplitude or kinetics [male amp: *t*(8) = 0.55, *p* = 0.60; female amp: *t*(9) = 0.69, *p* = 0.51; male vs. female amp: *t*(17) = 0.87, *p* = 0.40; male rise: *t*(8) = 1.42, *p* = 0.19; female rise: *t*(9) = 0.72, *p* = 0.49; male vs. female rise: *t*(17) = 0.29, *p =* 0.77; male decay: *t*(8) = 0.59, *p* = 0.57; female decay: *t*(9) = 0.21, *p* = 0.84; male vs. female decay: *t*(17) = 0.54, *p =* 0.60; (Fig. 3E-G)]. Interestingly, NE increased the PL5 sEPSC frequency similarly in both sexes [male: *t*(9) = 3.90, *p* < 0.01; female: *t*(9) = 2.84, *p* < 0.05; male vs. female: *t*(18) = 0.51, *p* = 0.61; (Fig. 3H)], but continued to have no effect on the other sEPSC properties [male amp: *t*(9) = 1.88, *p* = 0.09; female amp: *t*(9) = 1.30, *p* = 0.22; male vs. female amp: *t*(18) = 0.032, *p =* 0.97; male rise: *t*(9) = 0.50, *p* = 0.63; female rise: *t*(9) = 0.083, *p* = 0.94; male vs. female rise: *t*(18) = 0.23, *p =* 0.82; male decay: *t*(9) = 0.53, *p* = 0.61; female decay: *t*(9) = 0.21, *p* = 0.84; male vs. female decay: *t*(18) = 0.36, *p =* 0.72; (Fig. 3I-K)].

The reverse was observed in the infralimbic (IL) mPFC, where NE increased the sEPSC frequency similarly in IL2/3 of both sexes [male: *t*(9) = 3.06, *p* < 0.05; female: *t*(9) = 2.91, *p* < 0.05; male vs. female: *t*(18) = 0.25, *p* = 0.81; (Fig. 3L)], and had no effect on the other sEPSC properties [male amp: *t*(9) = 0.049, *p* = 0.96; female amp: *t*(9) = 1.43, *p* = 0.19; male vs. female amp: *t*(18) = 1.26, *p =* 0.22; male rise: *t*(9) = 1.33, *p* = 0.22; female rise: *t*(9) = 0.86, *p* = 0.41; male vs. female rise: *t*(18) = 0.41, *p =* 0.69; male decay: *t*(9) = 1.49, *p* = 0.17; female decay: *t*(9) = 1.47, *p* = 0.18; male vs. female decay: *t*(18) = 0.19, *p =* 0.85; (Fig. 3M-O)]. NE also increased the IL5 sEPSC frequency in male mice, but had no effect in female mice [male: *t*(8) = 2.47, *p* < 0.05; female: *t*(9) = 0.68, *p =* 0.52; male vs. female: *t*(16) = 2.53, *p* < 0.05; (Fig. 3P)]. There were no effects of NE on the sEPSC amplitude or kinetics [male amp: *t*(8) = 0.31, *p* = 0.76; female amp: *t*(8) = 0.73, *p* = 0.49; male vs. female amp: *t*(16) = 0.27, *p =* 0.79; male rise: *t*(8) = 0.089, *p* = 0.76; female rise: *t*(8) = 1.94, *p* = 0.09; male vs. female rise: *t*(16) = 1.10, *p =* 0.29; male decay: *t*(8) = 0.32, *p* = 0.76; female decay: *t*(8) = 1.41, *p* = 0.20; male vs. female decay: *t*(16) = 1.36, *p =* 0.19; (Fig. 3Q-S)]. Overall, these data indicate that NE recruited presynaptic mechanisms to enhance glutamate transmission broadly across the male mPFC; in contrast, in female mice, NE potentiation of glutamate release was restricted to prelimbic layer 5 and infralimbic layer 2/3.

### Atipamezole-induced NE release altered cognitive performance in both sexes

**Fig. 4:**
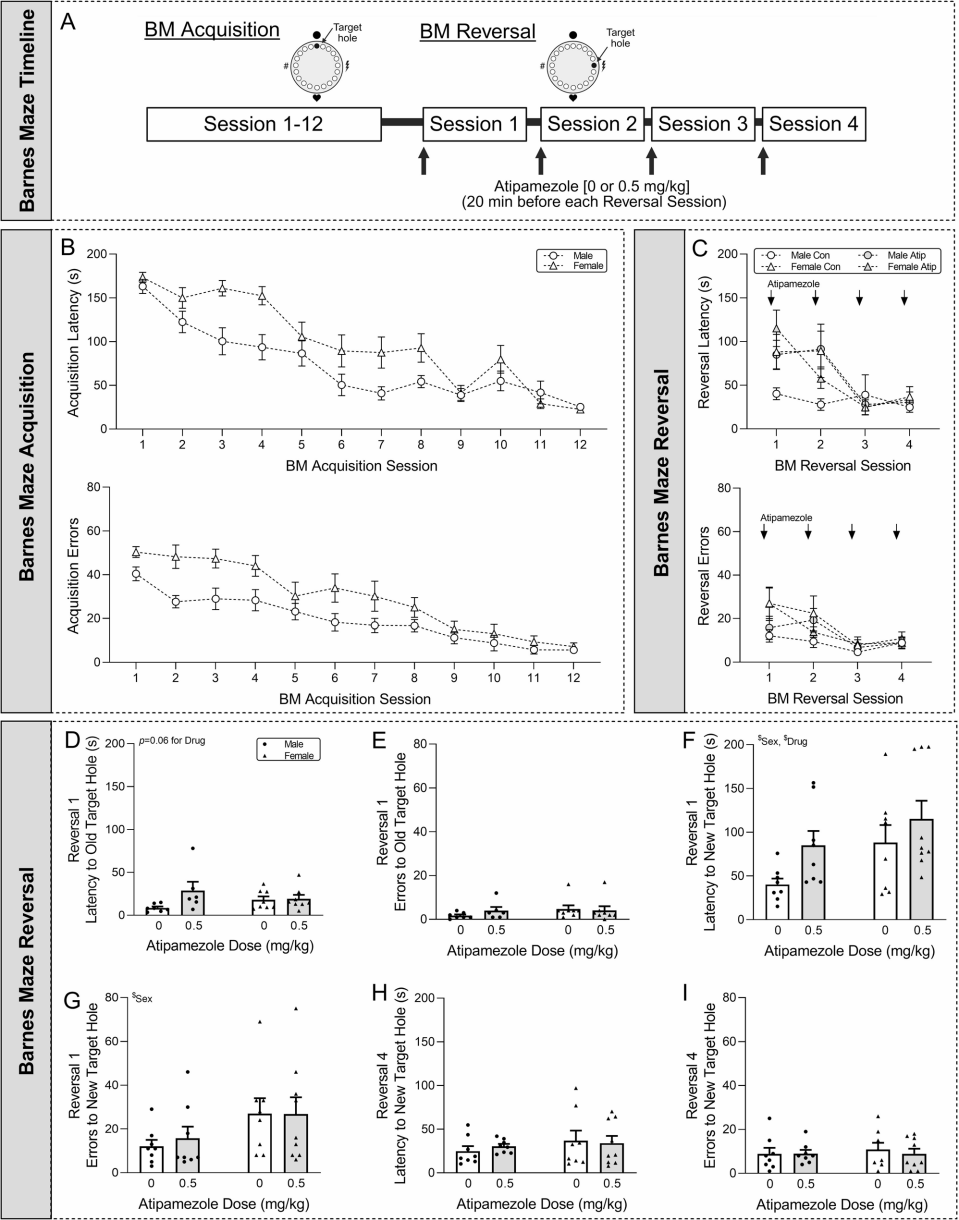
Atipamezole effects on behavioral flexibility. **A**. Schematic of the Barnes maze (BM) protocol used to assess the effects of atipamezole-induced NE release on behavioral flexibility. Atipamezole (0.5 mg/kg, *i.p.*) was administered 20 min before each reversal test. **B-C.** Time course of latency to enter the target hole and number of errors made prior to entering the target hole during (B) BM task acquisition and (C) BM reversal testing. **D-E.** During the first reversal test there were no significant differences in the (D) latency and (E) errors of mice approaching the old target hole location. **F.** During the first reversal test female mice took longer to find the new target hole location than males, and atipamezole impaired this latency in both sexes. **G.** During the first reversal test female mice made a greater number of errors prior to entering the new target hole than males. **H-I.** By the final reversal test there were no significant differences in the (H) latency and (I) errors of mice approaching the new target hole location. *N* = 8–9 mice per group. ^$^*p* < 0.05 by two-way ANOVA

To investigate how these sex differences in NE-induced glutamatergic signaling may shape mPFC-dependent behavioral flexibility we used the Barnes maze reversal task (Fig. 4A). In the final acquisition session (prior to any atipamezole administration), there were no sex differences in latency to enter the target hole or number of errors prior to target hole entry [latency: *t*(31) = 0.62, *p* = 0.54; errors: *t*(31) = 0.77, *p* = 0.45 by unpaired *t*-test; (Fig. 4B)]. The target hole was then moved to a new location and atipamezole (0.5 mg/kg *i.p.*) or vehicle was administered 20 min before each of the 4 reversal tests. During reversal 1, we first assessed the 24 h memory of each mouse by tracking their path to the old location of their target hole. Two-way ANOVA revealed that the main effect of drug approached significance for the latency, but there was no sex-by-drug interaction or main effect of sex [interaction: *F*(1,25) = 2.96, *p =* 0.10; sex: *F*(1,25) = 0.000004, *p* = 1.00; drug: *F*(1,25) = 3.77, *p =* 0.063 by two-way ANOVA; (Fig. 4C-D)]. There was also no difference in the number of errors [interaction: *F*(1,25) = 0.85, *p =* 0.37; sex: *F*(1,25) = 1.00, *p* = 0.33; drug: *F*(1,25) = 0.28, *p =* 0.60; (Fig. 4C, E)]. Of note, 1 Male Control, 2 Male Atipamezole and 1 Female Atipamezole mice did not travel to their old target hole so were excluded from this analysis. Barnes maze behavioral flexibility was then probed by tracking the path taken by mice to the newly-located target hole. During reversal 1, there were main effects of sex and drug on the latency to the new target hole [interaction: *F*(1,29) = 0.41, *p =* 0.53; sex: *F*(1,29) = 3.98, *p* = 0.055; drug: *F*(1,29) = 4.54, *p* < 0.05; (Fig. 4F)], indicating that females generally took longer to find the new location than males and that atipamezole impaired cognitive performance in both sexes. Females also made a greater number of errors compared to males at this timepoint [interaction: *F*(1,29) = 0.097, *p =* 0.76; sex: *F*(1,29) = 4.42, *p* < 0.05; drug: *F*(1,29) = 0.076, *p =* 0.78; (Fig. 4G)]. All of these effects were transient as by reversal day 4 there were no differences in the latency [interaction: *F*(1,29) = 0.28, *p =* 0.60; sex: *F*(1,29) = 0.96, *p* = 0.34; drug: *F*(1,29) = 0.028, *p =* 0.87; (Fig. 4H)] or errors [interaction: *F*(1,29) = 0.18, *p =* 0.68; sex: *F*(1,29) = 0.14, *p* = 0.71; drug: *F*(1,29) = 0.14, *p =* 0.71; (Fig. 4I)] to the new target hole.

**Fig. 5:**
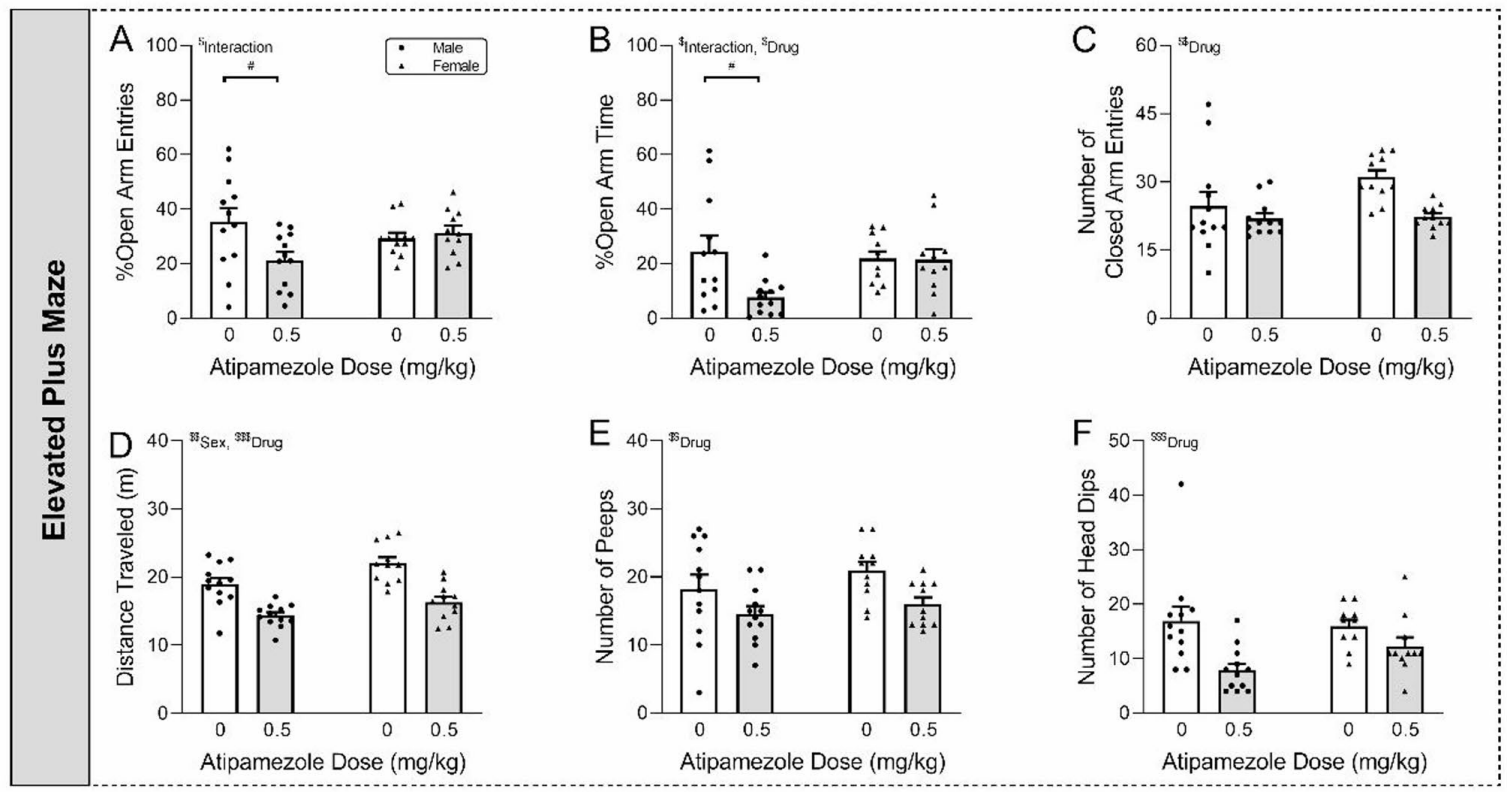
Atipamezole effects on anxiety-like behavior. **A-B.** Atipamezole (0.5 mg/kg, *i.p.*) administered 20 min before elevated plus maze (EPM) testing decreased the (**A**) percentage of entries and (**B**) percentage of time spent in the open arms in males. **C-D.** Atipamezole decreased the (**C**) number of closed arm entries and (D) total distance traveled in both sexes. There was also a main effect of sex on the distance traveled. **E-F.** Atipamezole decreased the number of (E) peeps and (F) head dips in both sexes. *N* = 11–12 mice per group. ^$^*p* < 0.05, ^$$^*p* < 0.01, ^$$$^*p* < 0.001 by two-way ANOVA, with ^#^*p* < 0.05 by *post hoc* Tukey’s multiple comparison test

Since the Barnes maze task is based on the innate inclination of mice to escape from a mildly aversive environment, we next assessed the effects of atipamezole (0.5 mg/kg *i.p.*) on anxiety-like behavior using the elevated plus maze (EPM). We found sex-by-drug interactions, with atipamezole decreasing the percentage of entries into the open arms [interaction: *F*(1,42) = 5.32, *p* < 0.05; sex: *F*(1,42) = 0.34, *p* = 0.56; drug: *F*(1,42) = 2.95, *p =* 0.093 by two-way ANOVA with *post hoc* Tukey’s test; (Fig. 5A)] and percentage of time spent in the open arms [interaction: *F*(1,42) = 4.39, *p* < 0.05; sex: *F*(1,42) = 2.03, *p* = 0.16; drug: *F*(1,42) = 4.97, *p* < 0.05; (Fig. 5B)] in males, suggesting increased anxiety-like behavior. Of note, atipamezole decreased general activity in both sexes as evidenced by the number of closed arm entries [interaction: *F*(1,42) = 2.46, *p =* 0.12; sex: *F*(1,42) = 3.08, *p* = 0.087; drug: *F*(1,42) = 9.08, *p* < 0.01; (Fig. 5C)], and total distance traveled during the task [interaction: *F*(1,42) = 0.47, *p =* 0.50; sex: *F*(1,42) = 10.22, *p* < 0.01; drug: *F*(1,42) = 43.62, *p* < 0.001; (Fig. 5D)]. There was also a main effect of sex on the distance traveled. Finally, atipamezole decreased exploratory peeps [interaction: *F*(1,42) = 0.17, *p =* 0.68; sex: *F*(1,42) = 2.02, *p* = 0.16; drug: *F*(1,42) = 8.26, *p* < 0.01; (Fig. 5E)] and head dips [interaction: *F*(1,42) = 2.25, *p =* 0.14; sex: *F*(1,42) = 0.95, *p* = 0.33; drug: *F*(1,42) = 13.11, *p* < 0.001; (Fig. 5F)] in both sexes.

**Fig. 6:**
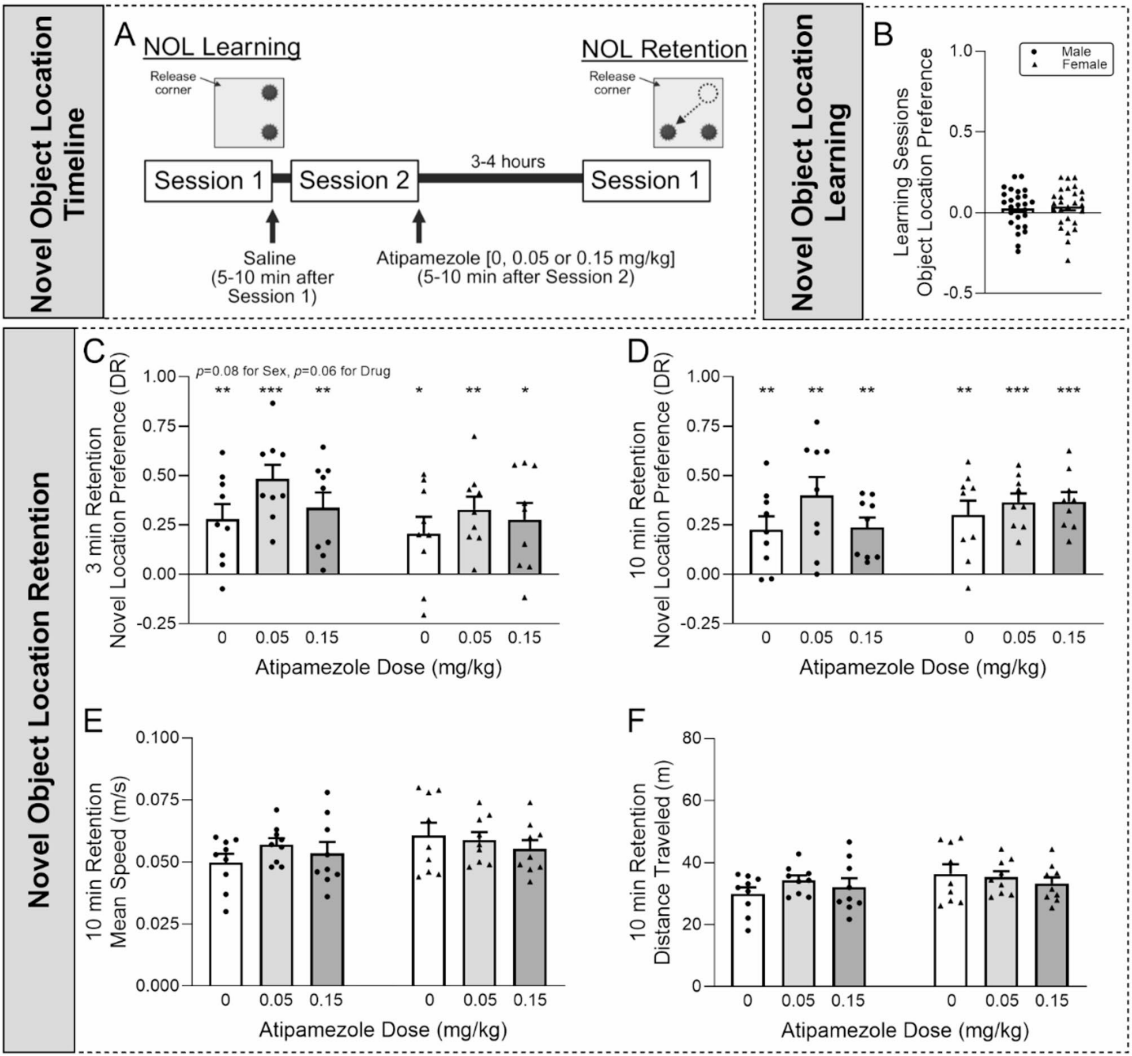
Atipamezole effects on episodic memory. **A**. Schematic of the novel object location (NOL) protocol used to assess the effects of atipamezole-induced NE release on episodic memory. Atipamezole (0.05 or 0.15 mg/kg *i.p.*) was administered during reconsolidation. **B.** Mice showed no object location preference during learning. **C.** In the first 3 min of the retention task, all groups showed increased preference for the object in the novel location. There were also trends towards significance supporting that males may show a greater preference towards the novel object location than females and that atipamezole may increase preference in both sexes. **D.** All groups continued to show increased preference for the object in the novel location over the 10-min duration of the task, but there were no group differences at this time point. **E-F.** Atipamezole did not alter the (E) mean speed and (F) total distance traveled in both sexes. *N* = 9 mice per group. *p < 0.05, **p < 0.01, ***p < 0.001 by one-sample *t*-test

Given these confounds, we decided to test the effect of lower doses of atipamezole on the mPFC-dependent novel object location task (NOL) to probe episodic memory (Fig. 6A). During learning (prior to any atipamezole administration), mice showed no object location preference [male: *t*(26) = 1.18, *p* = 0.25; female: *t*(26) = 1.56, *p* = 0.13 by one-sample *t*-test; (Fig. 6B)]. Atipamezole (0.05 or 0.15 mg/kg *i.p.*) or vehicle was then administered during the reconsolidation period between learning session #2 and the retention test. In the first 3 min of the retention task, all groups showed increased preference for the object in the novel location [male 0: *t*(8) = 3.68, *p* < 0.01; male 0.05: *t*(8) = 6.82, *p* < 0.001; male 0.15: *t*(8) = 4.40, *p* < 0.01; female 0: *t*(8) = 2.49, *p <* 0.05; female 0.05: *t*(8) = 4.98, *p <* 0.01; female 0.15: *t*(8) = 3.23, *p <* 0.05 by one-sample *t*-test; (Fig. 6C)], indicating intact episodic memory. Two-way ANOVA revealed main effects of sex and drug approached significance [interaction: *F*(2,48) = 0.19, *p =* 0.83; sex: *F*(1,48) = 3.22, *p* = 0.079; drug: *F*(2,48) = 2.91, *p =* 0.064], suggesting that males generally have a greater preference than females and that atipamezole may improve cognitive performance in both sexes. All groups continued to show increased preference for the object in the novel location over the 10-min duration of the task [male 0: *t*(8) = 3.40, *p* < 0.01; male 0.05: *t*(8) = 4.30, *p* < 0.01; male 0.15: *t*(8) = 4.74, *p* < 0.01; female 0: *t*(8) = 4.16, *p <* 0.01; female 0.05: *t*(8) = 8.29, *p <* 0.001; female 0.15: *t*(8) = 7.56, *p <* 0.001 by one-sample *t*-test; (Fig. 6D)], but there were no group differences at this time point [interaction: *F*(2,48) = 0.84, *p =* 0.44; sex: *F*(1,48) = 1.155, *p* = 0.29; drug: *F*(2,48) = 1.73, *p =* 0.19 by two-way ANOVA]. Interestingly, these doses of atipamezole did not impact locomotor function as there were no group differences in the mean speed [interaction: *F*(2,48) = 0.92, *p =* 0.41; sex: *F*(1,48) = 2.44, *p* = 0.13; drug: *F*(2,48) = 0.47, *p =* 0.63] or distance traveled [interaction: *F*(2,48) = 0.90, *p =* 0.42; sex: *F*(1,48) = 2.34, *p* = 0.13; drug: *F*(2,48) = 0.48, *p =* 0.62] during the 10 min task (Fig. 6E-F), in contrast to the higher atipamezole dose used in the Barnes maze experiment. Overall, these data indicate that while atipamezole bidirectionally impacted mPFC-dependent cognitive tasks (i.e. lower doses improved performance, while higher dose impaired it), there were no sex differences in its effects.

## Discussion

The PFC NE system plays a critical role in several stress-related neuropsychiatric diseases that manifest differently in men and women [2, 33]. However, most preclinical investigations of mPFC NE stress signaling and its behavioral outcomes have been conducted using only male subjects. This is particularly surprising as the LC is sexually dimorphic [1]. Thus, here we have taken a systems biology approach to probe NE regulation of the mPFC in both sexes. Most notably, we found that the LC→mPFC circuit was larger in female mice, and that subsets of female mPFC neurons were insensitive to NE (specifically, prelimbic mPFC layer 2/3 and infralimbic mPFC layer 5 neurons) compared to their male counterparts. These functional differences did not seem to alter NE’s effects on mPFC-dependent cognitive behaviors (reversal learning and episodic memory), though future studies should examine whether sex differences emerge with increased cognitive load as observed during periods of stress or alcohol withdrawal.

### Sexual dimorphism within the LC→mPFC NE system

It is well-established that women and female rodents have larger, more complex LCs than their male counterparts [1]. This may make the female LC more vulnerable to cognitive load and negative emotion, leading to greater brain-wide NE release and larger overall stress responses. Additionally, the mechanisms underlying LC activation are regulated in a sex-dependent manner, with female LC neurons activated by lower concentrations of corticotropin-releasing factor (CRF) [50], more responsive to acute stress through greater CRF_1_ receptor/G_s_-coupled recruitment of PKA/cAMP intracellular cascades [51] and less adaptable to excessive or repeated stress due to less CRF_1_ receptor/G_i_-coupled internalization by β-arrestin [51]. LC neurons form distinct circuits with different areas of the cortex [52, 53], and we found that a greater percentage of LC NE neurons project to the mPFC in female mice compared to males. While NE is released throughout the male mPFC, DβH+ fibers have denser varicosities in superficial vs. deep cortical layers [18]; it is currently unknown whether females show a similar pattern of NE inputs. Surprisingly, we found no difference in mPFC basal NE content between the sexes, though it is important to note that this measure does not reflect just basal extracellular NE levels or ongoing NE release as there can be significant amounts of NE stored in LC→mPFC terminals based on previous catecholamine studies [54, 55]. Another limitation of the current work is that brain NE levels dynamically shift based on a large variety of intersecting factors including age, circadian rhythm and sleep, appetite and hormone secretion, current and prior stress, and immune function [56–67]. Of particular relevance, female NE levels may be affected by estrous cycle; while cortical NE remained stable across the proestrus and diestrus phases and after ovariectomy or ovarian follicle depletion [68], others have found that estradiol treatment in ovariectomized rats can increase NE concentrations in the frontal cortex, hippocampus and hypothalamus [69–71]. Additionally, a *post mortem* study of human PFC tissue reported greater activity of NE degradation enzymes (i.e. catechol-O-methyltransferase; COMT) in men compared to women, though there was no difference in PFC *COMT* gene expression [72]. We also found no sex differences in total mPFC AR gene expression, though we did not probe for the β3 subtype and recent work has identified a novel role for it in the mPFC where it depolarizes layer 5 pyramidal neurons in male juvenile rats [21], potentially via increased NMDA receptor activity [73]. Another caveat is that previous studies have observed mPFC layer-specific AR patterning of glutamatergic vs. GABAergic neurons, though to the best of our knowledge potential sex differences have not been investigated [2]. Consequently, even small sex differences in NE release dynamics or layer-/cell type-specific AR expression could significantly alter its regulation of mPFC neurons.

We found that 1 µM NE broadly increased glutamate release onto mPFC pyramidal neurons in males, but in females the NE-induced increase was only observed in PL5 and IL2/3. Since NE was applied directly onto the recording neuron, we expect that these sex differences stem from input-specific AR expression in male vs. female PL2/3 and IL5 pyramidal neurons; while all AR subtypes are distributed throughout the male mPFC, α2R and α1R are more highly expressed in layers 2/3 and βR predominate in layer 5 [2, 74]. Moreover, each AR subtype also has differing NE binding affinity (α2R >α1R >βR) and G-protein coupling (α2R/G_i_-, α1R/G_q_-, or βR/G_s_-coupled), which can lead to concentration-dependent opposing effects on neuronal activity [2]. Regardless, our present findings agree with [28] who reported that 10 µM NE increased glutamate release and pyramidal cell firing in PL2/3 in male rats, as well as others reporting that NE increased mPFC glutamate transmission via either α1 or β receptors [31, 75]. Some studies have also found the opposite result [27, 29, 76]; in particular [27], observed that activation of α2 receptors increased GABA-mediated inhibition of male mPFC pyramidal neurons. In our electrophysiology experiments, we pharmacologically-isolated glutamate currents using GABA_A_ and GABA_B_ receptor antagonists, and so future work should explore potential sex differences in NE/AR influence over mPFC inhibitory microcircuits in a subregion- and layer-specific manner. This will help provide greater clarity about how our observed sex differences in NE responsivity in PL2/3 and IL5 impact the overall mPFC network, potentially leading to sex-specific activation of downstream regions like the ventral tegmental area, medial amygdala, lateral septum, nucleus accumbens core and periaqueductal gray [77–81].

### No sex differences in NE-mediated cognitive performance

Rising mPFC NE levels shift cognitive function from a state of relaxation to alertness and then to hyperarousal and cognitive impairment [2]. Accordingly, we found in both sexes that lower doses of atipamezole (0.05–0.15 mg/kg) improved episodic memory during the novel object location task, while a higher dose (0.5 mg/kg) impaired behavioral flexibility in the Barnes maze reversal task. Of note, the Barnes maze task is designed to be mildly aversive so that the mice are motivated to escape the platform, which could potentially further increase their mPFC NE levels. The higher dose of atipamezole also increased anxiety-like behavior only in males, perhaps due to enhanced NE function in the mPFC or other stress-sensitive regions like the central amygdala and bed nucleus of the stria terminalis [2, 33, 39, 82–85], as well as decreased exploratory and locomotor behavior in both sexes. Another potential limitation is that atipamezole may inadvertently trigger dopamine co-release from NE terminals [86] which can also contribute to mPFC aversive processing [87]. Regardless, other studies report similar results; atipamezole at low doses improved alertness, selective attention, planning, learning, and recall, while higher doses led to impaired cognitive function [44, 88–92]. While none of these NE-specific studies included female subjects, natural fluctuations in ovarian hormones have been shown to regulate learning strategies [93, 94], but not working, episodic or reference memory [95–97]. Males also tend to exhibit deficits in mPFC-dependent behaviors after stress exposure, while females show hyperarousal which can lead to extinction deficits and increased probability of developing PTSD-like symptoms [98]. Therefore, these sex-specific behavioral sequelae should be explored further in the context of mPFC NE signaling, heightened cognitive load and neuropsychiatric disease.

## Conclusions

Given the well-established sexual dimorphism of the LC NE system, the goal of this study was to investigate its regulation of basal mPFC function in both sexes. We found that female mice had a larger LC→mPFC circuit and that their prelimbic layer 2/3 and infralimbic layer 5 glutamate synapses were NE-insensitive compared to their male counterparts. These functional differences did not affect NE-induced alterations in mPFC-dependent cognitive performance, though future studies should examine whether sex differences emerge with increased cognitive load as observed during periods of stress or alcohol withdrawal [33]. This is particularly important as both stimuli increase cerebral spinal fluid and brain NE and its metabolites in humans [99–105], and preclinical studies have shown that the male LC habituates to repeated stress or alcohol exposures while the female LC does not [70, 106]. Clinically, women exhibit greater stress-induced activation of the NE system [107], are more likely to be diagnosed with affective disorders like posttraumatic stress disorder [108] and are more likely to drink alcohol to regulate negative affect and stress reactivity than men [109]. Therefore, our study highlights the importance of considering specific subpopulations (e.g. women, people with comorbid AUD and PTSD) during the ongoing development of new NE-based therapeutics to treat neuropsychiatric disorders.

## Data Availability

The datasets used and/or analyzed during the current study are available from the corresponding author on reasonable request.
